# Small heterodimer partner interacting leucine zipper protein (SMILE) ameliorates autoimmune arthritis via AMPK signaling pathway and the regulation of B cell activation

**DOI:** 10.1186/s12964-023-01054-y

**Published:** 2023-05-04

**Authors:** JooYeon Jhun, Jeonghyeon Moon, Ji Ye Kwon, Keun-Hyung Cho, Seang Yoon Lee, Hyun Sik Na, Mi-La Cho, Jun-Ki Min

**Affiliations:** 1 Rheumatism Research Center, College of Medicine, Catholic Research Institute of Medical Science, The Catholic University of Korea, Seoul, 06591 Korea; 2 Lab of Translational ImmunoMedicine, Catholic Research Institute of Medical Science, College of Medicine, The Catholic University of Korea, Seoul, 06591 Korea; 3 Department of Biomedicine and Health Sciences, College of Medicine, The Catholic University of Korea, 222, Banpo-Daero, Seocho-gu, Seoul, 06591 Republic of Korea; 4 Departments of Neurology and Immunobiology, Yale School of Medicine, New Haven, 06511 CT USA; 5 Department of Medical Life Sciences, College of Medicine, The Catholic University of Korea, 222, Banpo-daero, Seocho-gu, Seoul, 06591 Republic of Korea; 6 Department of Internal Medicine, The Clinical Medicine Research Institute of Bucheon St. Mary’s Hospital, Bucheon-si, South Korea

**Keywords:** Small heterodimer partner-interacting leucine zipper protein (SMILE), Rheumatoid arthritis (RA), B cell, BAFF receptor, AMPK/mTOR

## Abstract

Rheumatoid arthritis (RA) is an autoimmune disease that causes joint swelling and inflammation and can involve the entire body. RA is characterized by the increase of pro-inflammatory cytokines such as interleukin (IL) and tumor necrosis factor, and the over-activation of T lymphocytes and B lymphocytes, which may lead to severe chronic inflammation of joints. However, despite numerous studies the pathogenesis and treatment of RA remain unresolved. This study investigated the use of small heterodimer partner-interacting leucine zipper protein (SMILE) overexpression to treat a mouse model of RA. SMILE is an insulin-inducible corepressor through adenosine monophosphate-activated kinase (AMPK) signaling pathway. The injection of a SMILE overexpression vector to mice with collagen induced-arthritis resulted in a milder clinical pathology and a reduced incidence of arthritis, less joint tissue damage, and lower levels of Th17 cells and plasma B cells in the spleen. Immunohistochemistry of the joint tissue showed that SMILE decreased B-cell activating factor (BAFF) receptor (BAFF-R), mTOR, and STAT3 expression but increased AMPK expression. In SMILE-overexpressing transgenic mice with collagen antibody-induced arthritis (CAIA), a decrease in the arthritis score and reductions in tissue damage, the number of B cells, and antibody production were observed. The treatment of immune cells in vitro with curcumin, a known SMILE-inducing agent, led to decreases in plasma B cells, germinal center B cells, IL-17-producing B cells, and BAFF-R-positive B cells. Taken together, our findings demonstrate the therapeutic potential of SMILE in RA, based on its inhibition of B cell activation mediated by the AMPK/mTOR and STAT3 signaling pathway and BAFF-R expression.

**Video abstract**

**Supplementary Information:**

The online version contains supplementary material available at 10.1186/s12964-023-01054-y.

## Introduction

Small heterodimer partner-interacting leucine zipper protein (SMILE) is an isoform of the transcription factor cyclic AMP-response element binding protein Zhangfei (CREBZF) [1]. CREBZF is a member of the activating transcription factor/cAMP response element binding protein (ATF/CREB) family that regulates various cellular functions, including transcription [2–4]. SMILE is an insulin-inducible corepressor that acts by decreasing PGC-1α expression [5], hepatic glucogenesis [6], and adenosine monophosphate-activated kinase (AMPK) signaling [7]. Despite its many roles in cells, the underlying mechanism and role of SMILE in immune cells are unknown. In this study, a mouse model of rheumatoid arthritis (RA) was used to investigate the effect of SMILE expression on immune cells.

RA is a systemic chronic autoimmune disease characterized by synovitis, increases in pro-inflammatory cytokines such as interleukins (ILs) and tumor necrosis factor (TNF), and the overactivation of T and B lymphocytes [8, 9]. Although RA does not significantly affect survival, it causes chronic pain following the erosion and destruction of cartilage, bone, and tendons [10]. The pathogenesis of RA is complex and no single drug is effective in its treatment completely; many patients are unresponsive, including to methotrexate [11]. Accordingly, more effective control for RA is needed.

Many previous studies of RA have focused on the role of T lymphocytes [12–14]. Activated T helper (Th)1, Th17, and autoreactive CD4^+^ T cells have been implicated in the pathogenesis of RA together with pro-inflammatory cytokines secreted by T cells, including IL-6, IL-17, IL-23, and TNF [15–18]. However, B cells play an equally important role in RA, as their accumulation in tertiary lymphoid tissues (TLTs) is related to higher RA pathogenic scores and T cell activation in RA patients [19–21]. Among the chemokines and inflammatory factors expressed by TLTs are LTα, LTβ, CXCL13, CCL20, CCL21, and CXCL12, all of which induce inflammation and the infiltration of inflammatory cells into joints [22–25]. The production of autoantibodies by B cells also contributes to the pathogenesis of RA [21, 26, 27]. For example, anti-citrullinated protein antibodies are commonly detected in patients with RA and are the focus of current interest in approaches based on targeted therapy [28].

This study investigated the potential therapeutic effect of SMILE expression in a mouse model of RA. Mice with collagen-induced arthritis (CIA) were injected with a SMILE overexpression vector and SMILE overexpression transgenic mice were used for collagen antibody-induced arthritis (CAIA). Besides, the effect of curcumin (diferuloylmethane) in CIA mice was determined as well, as a previous study showed that it induces SMILE expression via AMPK signaling and the suppression of endoplasmic reticulum stress-responsive gene transcription. Therefore, we treated curcumin with splenocytes and analyzed the proportion of immune cells.

Our results showed that SMILE expression inhibits B cell activation and differentiation in mouse models of RA, thus demonstrating its potential efficacy as a therapeutic regulator in autoimmune diseases such as RA.

## Materials & methods

### Animals

Seven-week-old male DBA/1 J mice (Orient Bio, Gyeonggi-do, Korea) were purchased from Orient Bio Inc. (South Korea). The animals were maintained under specific pathogen-free conditions in an animal facility with controlled humidity (55 ± 5%), light (12 h/12 h light/dark), and temperature (22 ± 1 °C). The air in the facility passed through a HEPA filter system designed to exclude bacteria and viruses. Animals were fed mice chow and water ad libitum. All experimental procedures were provided in accordance with the Laboratory Animals Welfare Act, the Guide for the Care and Use of Laboratory Animals and the Guidelines and Policies for Rodent experiment provided by the IACUC (Institutional Animal Care and Use Committee) in school of medicine, The Catholic University of Korea. To generate SMILE transgenic mice, a pcDNA3.0 vector was constructed containing a cytomegalovirus promoter. The SMILE fragment was synthesized by GenScript Corporation (USA), with codon optimization for expression in mammalian cells. The open reading frame originated in mice. Transgenic mice overexpressing SMILE were generated on a C57BL/6 background by transgene microinjection and maintained at Macrogen Inc. (South Korea). SMILE transgenic founder mice were mated to C57BL/6 mice and were crossed for 10 generations. The presence of the transgene in the founders was confirmed by polymerase chain reaction (PCR) using genomic DNA extracted from the tail. All experimental procedures were evaluated and conducted in accordance with the protocols approved by the Animal Research Ethics Committee at the Catholic University of Korea (Permit Number: 2021-0010-01, 2020-0306-02)**.**

### Induction and assessment of collagen induced arthritis (CIA) and SMILE administration

To induce CIA, 0.1 mL of emulsion containing 100 μg of bovine type II collagen (CII) and Freund’s complete adjuvant (Chondrex, WA, USA) and Freund’s complete adjuvant (Chondrex) was injected intradermal into the base of the tail as a primary immunization. Two weeks later, a booster injection of 100 μg CII dissolved and emulsified 1:1 with water, a booster Freund’s incomplete adjuvant (Difco, MI, USA) was administered into the tail. At 1 week after collagen-induced arthritis (CIA) induction, SMILE mock control vector was administered by electroporation; weekly hydrodynamic injection was then performed for 10 weeks, as described previously. SMILE overexpression experiments, SMILE cDNA fragment was cloned into the pcDNA3.0 vector between EcoRI and KpnI. *Escherichia coli* containing the SMILE overexpression vector were incubated in Luria–Bertani high salt broth (Duchefa Biochemie, Netherlands) for 16 h at 37 °C and 160 rpm. The cells were harvested by centrifugation, the SMILE overexpression vector purified using a NucleoBond Xtra Maxi EF Kit (Macherey–Nagel, Germany). Mice were intravenously injected with 100 μg of SMILE overexpression vector or control vector in 1 mL of saline.

### Induction and assessment of collagen antibody-induced arthritis (CAIA)

Eight-week-old male C57BL/6 mice and SMILE transgenic mice were used for the induction of CAIA. Anti-collagen antibodies 2 mg (Chondrex) were injected intravenously in to 2 groups of mice, and 3 days later, 50 μg of LPS was administered intraperitoneally. Mice were monitored and evaluated daily for arthritis.

### Clinical assessment of arthritis

The severity of arthritis was evaluated by three independent observers. The mice were observed twice weekly or daily to determine the onset and severity of joint inflammation for up to 7 weeks after the primary immunization. The severity of arthritis was assessed on a scale of 0–4, based on the following criteria: 0 = no edema or swelling 1 = slight edema, with erythema with erythema limited to the foot or ankle; 2 = slight edema, with erythema from the ankle to the tarsal bone; 3 = moderate edema, with erythema from the ankle to the tarsal bone; and 4 = severe edema, with erythema from the ankle to the entire leg. The arthritis score of each mouse was calculated as the sum of the scores of the four limbs; the highest possible arthritis score for each mouse was 16. The mean arthritis index was used to compare the scores of the control and experimental groups [29].

### Histological analysis

Mouse joint tissues were fixed in 4% paraformaldehyde (Sigma-Aldrich, St. Louis, MO, USA), decalcified in a histological decalcifying solution (Calci-Clear Rapid; National Diagnostics, Atlanta, GA, USA), and embedded in paraffin wax for histological analyses. Sections (7 mm) were prepared and stained with hematoxylin (YD Diagnostics, Yongin, Korea), eosin (Muto Pure Chemicals Co., Ltd., Tokyo, Japan), and safranin O (Sigma-Aldrich) [30].

### Immunohistopathological analysis of arthritis

Joint tissues were incubated overnight at 4 °C with primary antibodies against SMILE (Abcam, Cambridge, UK), B-cell activating factor receptor (BAFF-R) (Abcam), pAMPK (Abcam), mTOR (Cell Signaling, MA, USA), pSTAT3 ser727 (Abcam), Pstat3 Tyr705 (Abcam), IL-1β (Abcam), IL-6 (Abcam), IL-17(Abcam). Subsequently, samples were incubated with a biotinylated streptavidin–peroxidase complex for 1 h, and the signals were developed using chromogen 3,3′- diaminobenzidine (Thermo Scientific, Rockford, IL, USA). The sections were examined under a photomicroscope (Olympus, Tokyo, Japan). The number of positive cells in high-power digital images (magnification, ×400) was counted using Adobe Photoshop software (Adobe, San Jose, CA, USA). Stained cells were counted independently by three observers, and the mean values were evaluated.

### Immunofluorescence imaging

Th17 (CD4, IL-17), Treg (CD4, CD25, Foxp3), SMILE, AMPK, mTOR, pSTAT3 705, pSTAT3 727 and germinal center markers (CD3, B220, PNA) expression levels were analyzed by confocal microscopy. Tissue Sects. (7 μm thick) were fixed in methanol-acetone and stained with phycoerythrin (PE)-conjugated anti-CD4 (Biolegend, San Diego, CA, USA), fluorescein isothiocyanate (FITC)-conjugated anti CD4 (BD Bioscience, San Diego, CA, USA), PE-conjugated anti-interleukin-17 (eBioscience, San Diego, CA, USA), allophycocyanin (APC)-conjugated anti-CD25 (Biolegend), PE-conjugated anti-Foxp3 (Thermo Fisher Scientific, Waltham, MA, USA), PE-conjugated anti-phosphorylated Stat3 (Tyr 705, Ser 727, BD Bioscience), APC-conjugated anti-B220 (eBioscience), Alexa 594-conjugated anti-PNA (Thermo Fisher Scientific), SMILE (Abcam, Cambridge, UK), AMPK (Abcam) and mTOR (Cell Signaling). After overnight incubation at 4℃, the stained sections were analyzed on a Zeiss confocal microscope (LSM 700; Carl Zeiss, Oberkochen, Germany) at 200× magnification and Zen Blue edition software.

### Western blotting

Cells were lysed in RIPA lysis and extraction buffer containing Halt protease inhibitor cocktail (Thermo Scientific, USA). Lysates were centrifuged at 14,000 rpm for 15 min at 4 °C. Protein concentration was determined using a Pierce BCA Protein Assay Kit (Thermo Scientific, USA). Proteins were resolved by sodium dodecyl sulfate–polyacrylamide gel electrophoresis and transferred to membranes (GE Healthcare, USA). The membranes were incubated with antibodies against SMILE, phosphorylated AMPK (Cell Signaling, MA, USA), SMILE, GAPDH (Abcam, Cambridge, UK). Hybridized bands were detected by enhanced chemiluminescence (Thermo Scientific, USA) on X-ray film (AGFA, Belgium). Western blotting was performed using the SNAP i.e. Protein Detection System.

### Isolation and stimulation of splenocytes and culture

Splenocytes were prepared from the spleens of Collagen induced arthritis mice. Splenocytes were maintained in Roswell Park Memorial Institute (RPMI)-1640 medium supplemented with 5% fetal bovine serum (Gibco, Grand Island, NY, USA). Splenocytes were stimulated with or Curcumin (Sigma-Aldrich) 25 μg and LPS (Sigma-Aldrich) 100 ng/mL for 12 h and 72 h; they were then subjected to western blot analysis and flow cytometry, respectively.

### Flow cytometry

The spleens were removed from the mice and single cells were isolated and stained using fluorescently conjugated antibodies against Follicular B and Marginal B cells; APC-conjugated anti-B220 (eBioscience), Percp/Cy5.5-conjugated CD23 (Biolegned) and Pacific Blue-conjugated CD21 (Biolegend), Mature B and Immature B cells; APC-conjugated anti-B220 (eBioscience), FITC-conjugated anti IgD (Thermo Fisher Scientific) and PE-conjugated anti-IgM (Thermo Fisher Scientific), regulatory B cells; FITC-conjugated anti-CD5 (Invitrogen), PE-conjugated CD1d (eBioscience) and PE/Cy7-conjugated anti-CD19 (BD Bioscience), Plasma B; ACP-conjugated anti-B220 (eBioscience) and PE-conjugated anti-CD 138 (BD Bioscience), Germinal center B; APC-conjugated anti-B220 (eBioscience) and Alexa Flour 488-conjugated anti-GL7 (Biolegend), IL-17 secreting B; PE/Cy7-conjugated anti-CD19 (BD Bioscience) and PE-conjugated anti-interleukin-17 (eBioscience) and BAFF-R; PE/Cy7-conjugated anti-CD19 (BD Bioscience) and PE-conjugated anti-CD268 (Biolegend). Prior to intracellular staining, cells were stimulated for 4 h with phorbol 12-myristate 13-acetate (PMA) (25 ng/mL) and ionomycin (250 ng/mL) in the presence of GolgiStop™ (BD Biosciences, USA). Intracellular staining was performed using a BD Cytofix/Cytoperm Plus Fixation/Permeabilization Kit and BD Golgistop Kit (BD Biosciences, USA). The transcription factor Foxp3 was stained using a Foxp3/Transcription Factor Staining Kit (eBioscience, USA) according to the manufacturer’s instructions. Flow cytometry was performed using a cytoFLEX Flow Cytometer (Beckman Coulter, USA).

### Measurement of immunoglobulin

Serum was gathered when sacrificed for concentration of Total IgG, IgG1 and IgG2a, and the serum was stored at – 70 °C until further use. The levels of the Total IgG, IgG1 and IgG2a antibodies in the serum were measured using ELISA (Bethyl Laboratories, Montgomery, TX, USA).

### Statistical analysis

Statistical analysis was performed using Prism9 (GraphPad Software, USA). Normally distributed continuous data were analyzed by parametric Student’s t-test. Differences in means among groups were subjected to analysis of variance followed by Bonferroni post hoc test. Values are means ± standard error of the mean (SEM). A value of *P* < 0.05 was indicative of statistical significance.

## Results

### SMILE overexpression inhibits RA pathogenesis in a CIA mouse model

**Fig. 1:**
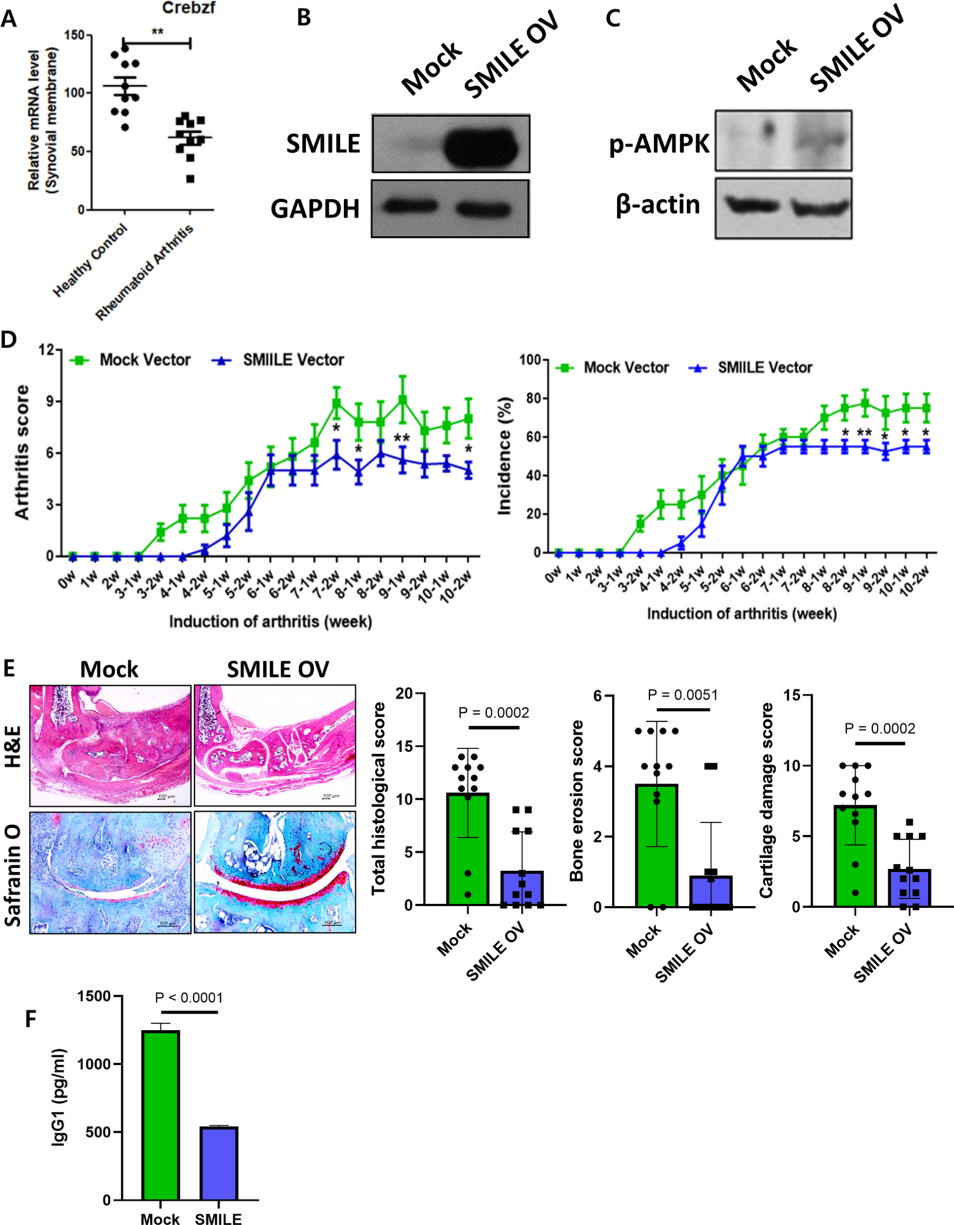
SMILE overexpression attenuates RA pathogenesis in Collagen Induced Arthritis (CIA) mouse model. **A** The expression of SMILE which is coded by Crebzf in PBMCs from RA patients and healthy controls. **B**, **C** Lysates of NIH3T3 cells transfected with a mock vector or the SMILE vector were analyzed for SMILE and phosphorylated AMPK (p-AMPK) expression using western blotting. **D** CIA was induced in DBA1/J mice. On days 7 and 21 post immunization, mock or SMILE overexpression vectors were hydro dynamically injected. Vectors were also administered in the thigh area and transfected via electroporation once every 10 days until the end of the experiment. Arthritis scores were determined at different time points during the experimental period. **E** Tissue sections of the joints of mice treated with mock vector or the SMILE vector were immunostained with H&E (bar = 100 µm) and safranin O (bar = 100 µm), and the histologic score for features of arthritis were determined. The dots of bar graph represent 1 mouse respectively. **F** Concentrations of type II collagen (CII)-specific total IgG and IgG2a as determined by enzyme-linked immunosorbent assays. The results are the mean ± SD of three independent experiments. Data are presented as the *P < 0.05 and **P < 0.01

The SMILE mRNA levels in peripheral blood mononuclear cells (PBMCs) from healthy controls and RA patients were examined using information contained in the National Center for Biotechnology Information Gene Expression Omnibus database. In our analysis, SMILE expression was significantly lower in RA patients than in healthy controls (Fig. 1A), suggesting a positive effect of SMILE in alleviating the symptoms of RA. We therefore investigated the effect of SMILE overexpression in a mouse model of RA. In RA mice injected with a SMILE overexpression vector (SMILE OV), SMILE and phosphorylated AMPK (p-AMPK) levels were significantly increased compared to the levels in control mice (Mock) (Fig. 1B and C). Measurements of the arthritis score and incidence in SMILE OV mice and Mock mice for 11 weeks showed that both values were significantly decreased in the SMILE mice (Fig. 1D). In hematoxylin and eosin (H&E)- and safranin O-stained joint tissue sections, The histological score, bone erosion score and cartilage damage score of SMILE OV were significantly decreased (Fig. 1E). In addition, IgG1 levels of serum were reduced by the overexpression of SMILE (Fig. 1F). This result showed that the SMILE overexpression vector was seen to have a protective effect on joint damage in RA mice.

### Modification of the BAFF receptor (BAFF-R), p-AMPK, mTOR, and STAT3 expression in SMILE mice

**Fig. 2:**
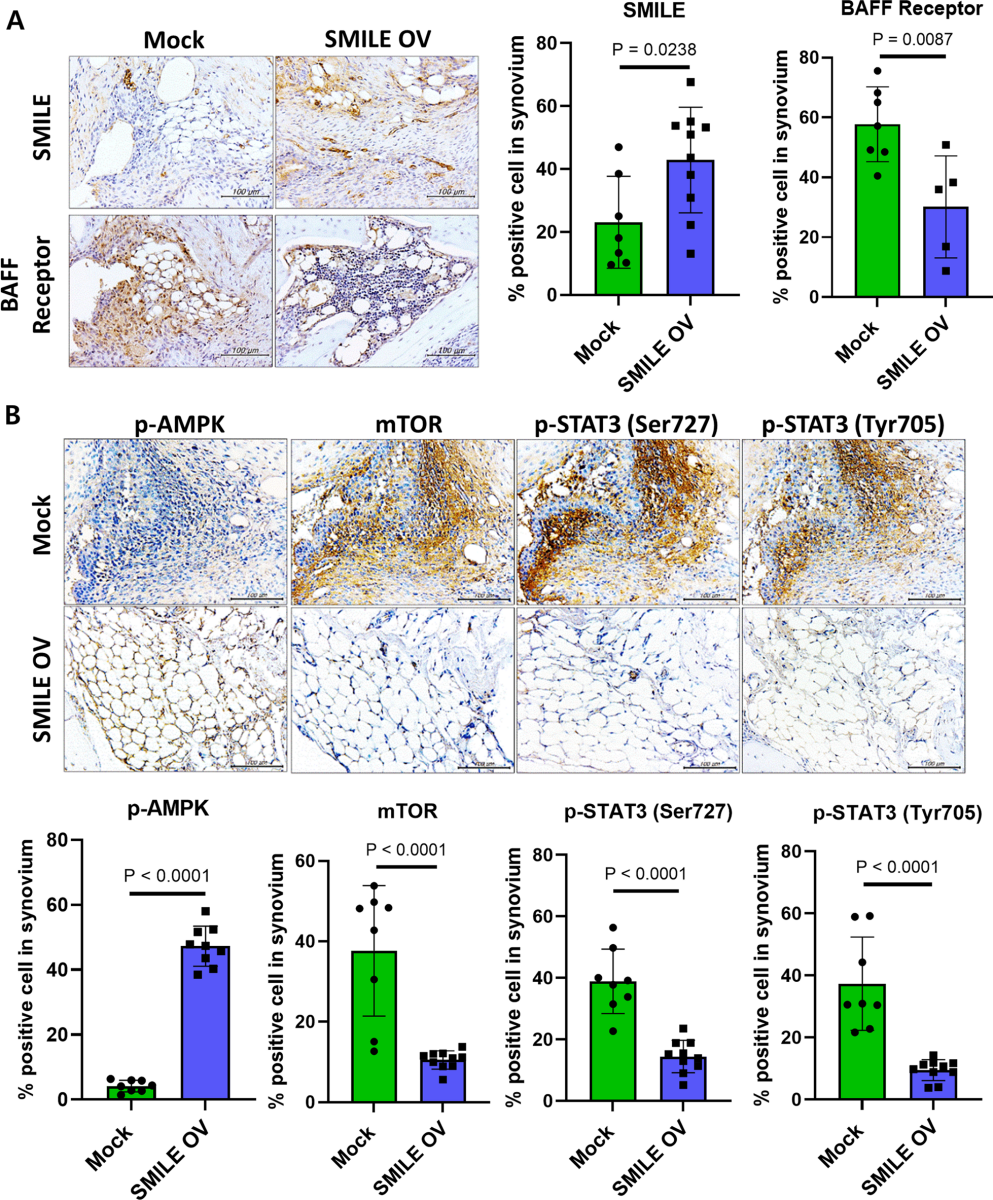
SMILE decreases BAFF-R, p-AMPK, mTOR, and STAT3 expression in CIA mice. Immunohistochemical staining of the joints of DBA1/J mice with CIA injected or not with the SMILE vector. **A** Tissue sections of mouse joints immunohistochemically stained for SMILE and BAFF-R. **B** Staining for anti-pAMPK, mTOR, pSTAT3 (ser727), and pSTAT3 (Thy705). Bars show the percentages of positive areas per field. Every dots represent 1 mouse respectively

To identify the signaling pathway by which SMILE expression suppresses RA symptoms, the joint tissues of RA mice were examined by immunohistochemistry. In SMILE OV mice, SMILE expression was elevated and the expression of BAFF-R in joint tissue was significantly reduced (Fig. 2A). Several signaling pathways (p-AMPK, mTOR, and STAT3) related to the production of pro-inflammatory cytokines were investigated as well, which showed the increased expression of p-AMPK and decreased expression of mTOR and STAT3 in SMILE OV mice compared to Mock mice (Fig. 2B). This result suggested that SMILE protein expression can ameliorate RA symptoms by reducing BAFF-R and regulating the levels of immune cells and thus of cytokines via the AMPK/mTOR signaling and STAT3 pathways.

### SMILE expression regulates the immune cell population

**Fig. 3:**
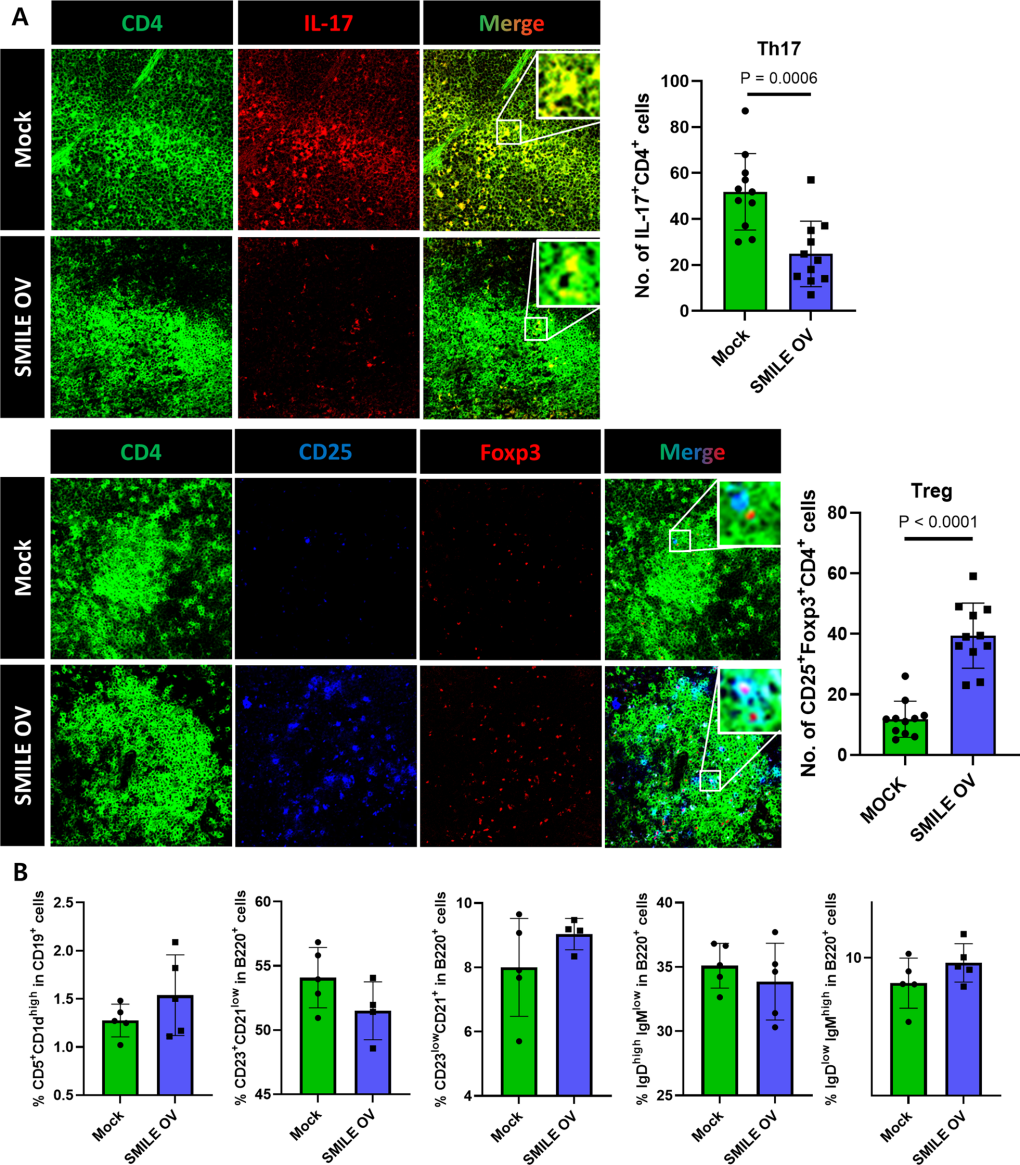
SMILE regulates the proportions of Th17 and Treg cells in CIA mice treated with the SMILE vector. **A** Immunofluorescence data of spleen cryosections from CIA mice administered a mock or SMILE vector using confocal microscopy. The sections were stained for CD4 and IL-17 (Th17 cells) or CD4, CD25, and Foxp3 (Treg cells) (original magnification, 200×). **B** Flow cytometry data of FoB cells (CD23^+^CD21^low^ in B220 cells), MZB cells (CD23^low^CD21^+^ in B220 cells), mature B cells (IgD^high^ IgM^low^ in B220 cells), immature B cells (IgD^low^ IgM^high^ in B220 cells), and B regulatory cells (CD5^+^CD1d^high^ in CD19 cells) in CIA mice administered a mock or SMILE vector. Every dots represent 1 mouse respectively

The ability of SMILE to regulate immune cells was examined by immunofluorescence staining of mouse spleen tissue. The results showed that SMILE overexpression decreased the number of Th17 cells (CD4^+^ IL-17^+^ cells) and increased the number of regulatory T cells (Treg; CD4^+^ CD25^+^ Foxp3^+^ cells) (Fig. 3A). Although not significant, there were also changes in the populations of regulatory B (Breg; CD5^+^ CD1D^high^ CD19^+^) cells, follicular B (FoB; CD23^+^ CD21^low^ B220^+^) cells, marginal zone B (MZB; CD23^low^ CD21^+^ B220^+^) cells, mature B (IgD^high^ IgM^low^ B220^+^) cells, and immature B (IgD^low^ IgM^high^ B220^+^) cells (Fig. 3B). These data were consistent with the regulation of immune cell populations by SMILE.

### Anti-inflammatory effect on CAIA in SMILE-overexpressing transgenic mice

**Fig. 4:**
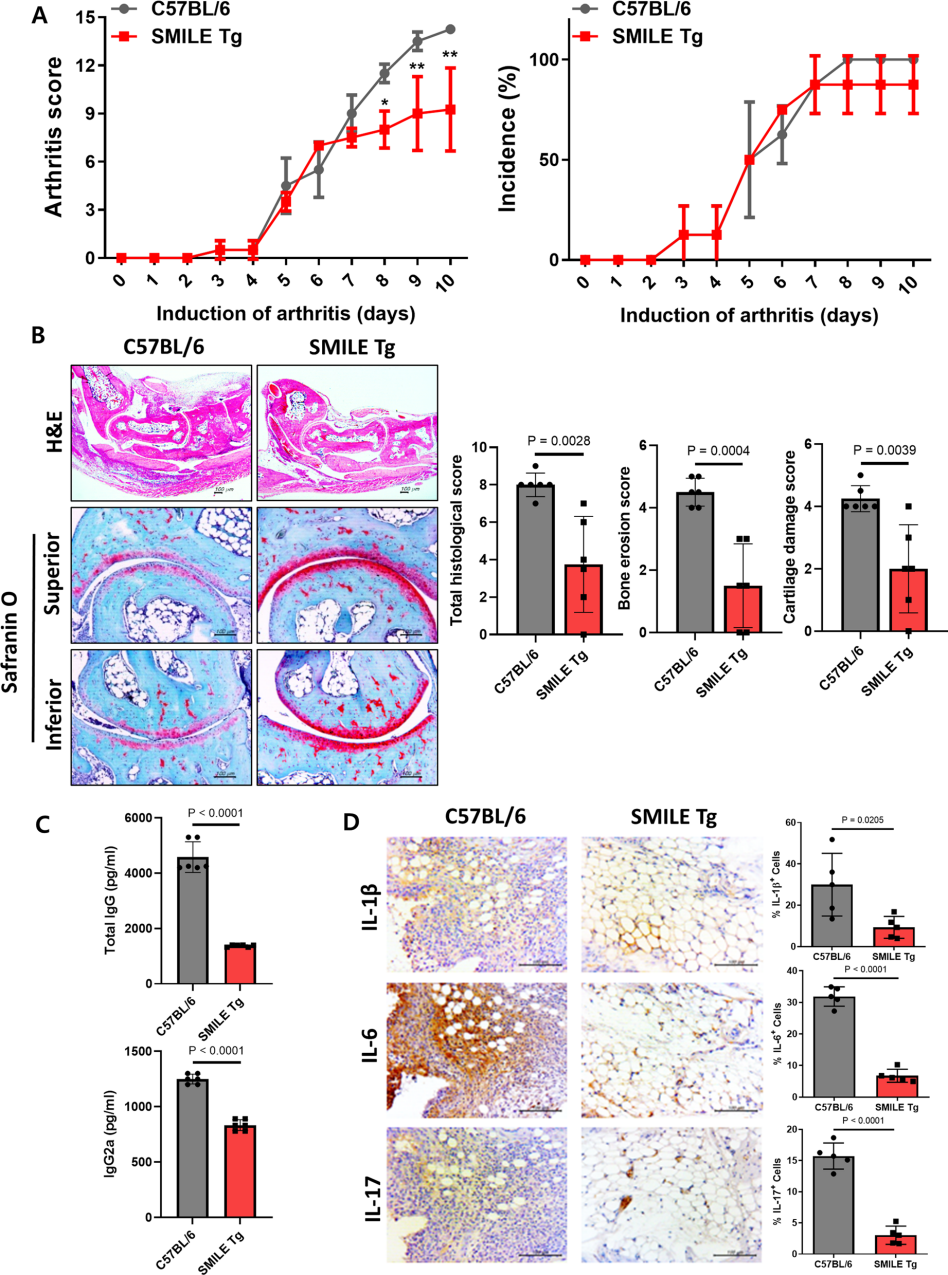
Attenuation of RA pathology in Collagen Antibody-Induced Arthritis (CAIA) SMILE transgenic (SMILE Tg) mice. CAIA was induced in wild-type (WT) C57BL/6 mice and SMILE Tg mice. **A** The severity of symptoms in each paw was scored daily on a scale from 0 (no symptoms) to 4 (maximal level of symptoms) and the scores for four limbs were summed. **B** Ten days after CAIA induction, tissue sections form the paw and ankle joints of the mice were stained with H&E. Representative histological features are shown. The quantified levels of inflammation, bone damage, and cartilage damage are shown in the graphs. **C** The levels of total IgG and IgG2a antibodies in the serum of CAIA mice 10 days after the first immunization. **D** Representative immunohistochemistry images showing that SMILE overexpression alleviated RA in CAIA mice. SMILE overexpression inhibited the expressions of IL-1β, IL-6, and IL-17 in CAIA mouse joints. Synovial sections from C57BL/6 and SMILE Tg mice stained for IL-1β, IL-6, and IL-17. Scale bar = 100 µm. *P < 0.05 and **P < 0.01. Every dots represent 1 mouse respectively

To investigate whether the effect of vector-mediated SMILE upregulation persisted, transgenic mice overexpressing SMILE (SMILE Tg) were established based on C57BL/6. Control (C57BL/6) and SMILE Tg mice were induced with CAIA. The arthritis scores and incidences of the SMILE Tg mice were lower than those of control mice (Fig. 4A). H&E and safranin O staining showed less histological damage, bone erosion and cartilage damage in SMILE Tg mice than in control C57BL/6 mice (Fig. 4B), as well as decreases in the total IgG and IgG2a levels (Fig. 4C). Immunohistochemistry of pro-inflammatory cytokine expression in mouse joint tissues showed that the IL-1β, IL-6, and IL-17 levels were significantly decreased in SMILE Tg mice (Fig. 4D), thus demonstrating the anti-inflammatory effect of SMILE on arthritis in SMILE Tg mice.

### SMILE-mediated inhibition of plasma B cell development and germinal center (GC) B cells

**Fig. 5:**
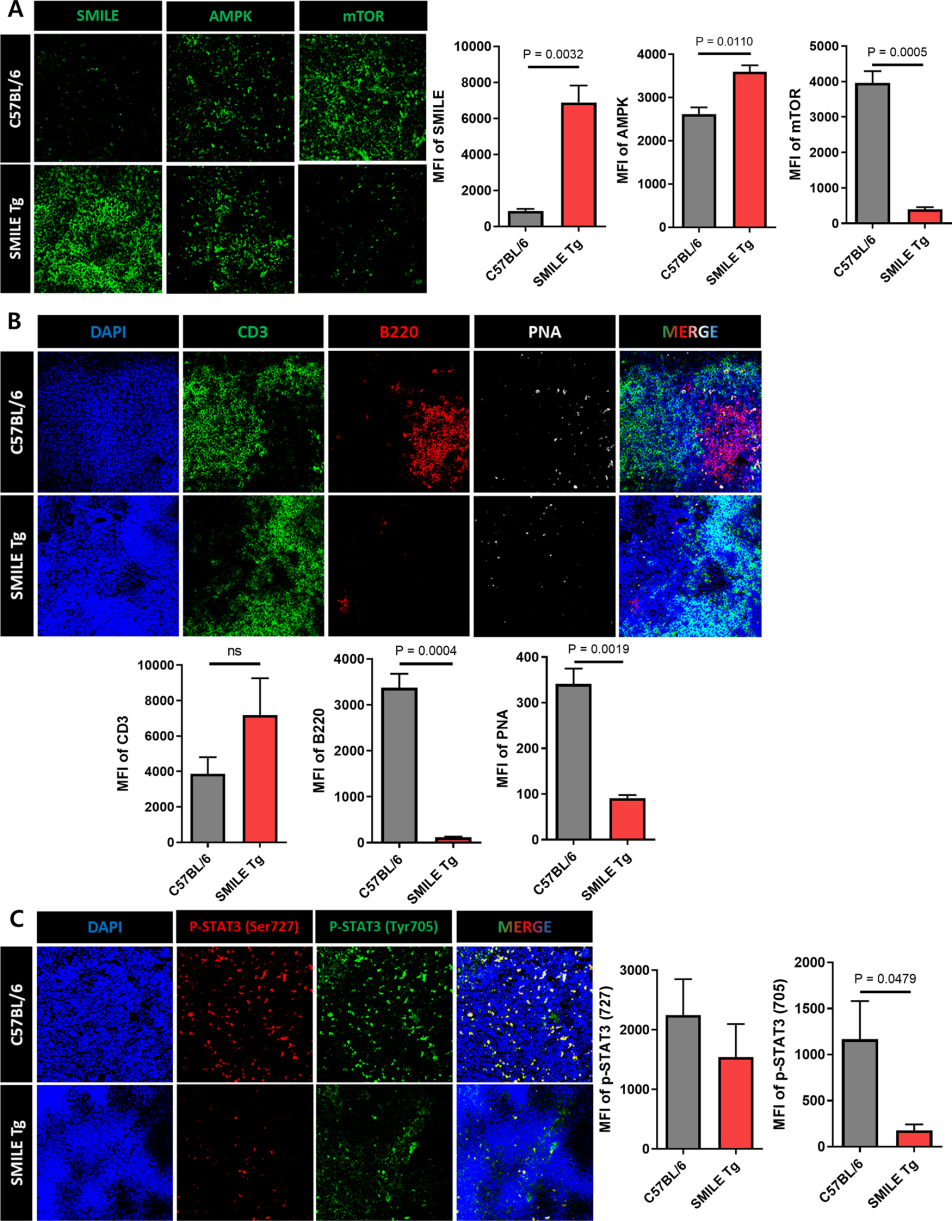
Effect of SMILE on germinal center (GC) B cell formation and signaling pathway in the spleen tissue of SMILE Tg mice. **A** The median fluorescence intensity (MFI) of SMILE, AMPK, and mTOR in CAIA C57BL/6 mice spleen and SMILE Tg mice spleen respectively. **B** B220^+^PNA^+^ GC B cells was analyzed by immunofluorescence. There was no significant difference in CD3, however, the expression of B220 and PNA was significantly decreased in SMILE Tg. **C** The phosphorylated (pSTAT3) 727 and pSTAT3 705 expression were decreased in SMILE Tg mice. Original magnification in 200×

Immunofluorescence was performed on mouse spleen tissue to identify the cells and pathways modified in SMILE Tg mice. First, the significantly increased expression of SMILE in SMILE Tg mice was confirmed. It was then shown that while AMPK expression was increased in SMILE Tg mice, mTOR expression was notably decreased in the SMILE Tg mice (Fig. 5A). Although CD3, a T cell marker, has no significant difference between C57BL/6 mice and SMILE Tg mice, the significantly reduced expression of B220, a B cell marker, and PNA, a germinal center (GC) B cell marker, was also found in the spleens of SMILE Tg mice (Fig. 5B). Also, the phosphorylated STAT3 (p-STAT3) expression was decreased in SMILE Tg mice (Fig. 5C). These results showed that SMILE inhibits the development of B cells by decreasing the activities of the AMPK/mTOR and STAT3 signaling pathways, thereby suppressing the progression of arthritis.

### Curcumin inhibits the activation of B cells

**Fig. 6:**
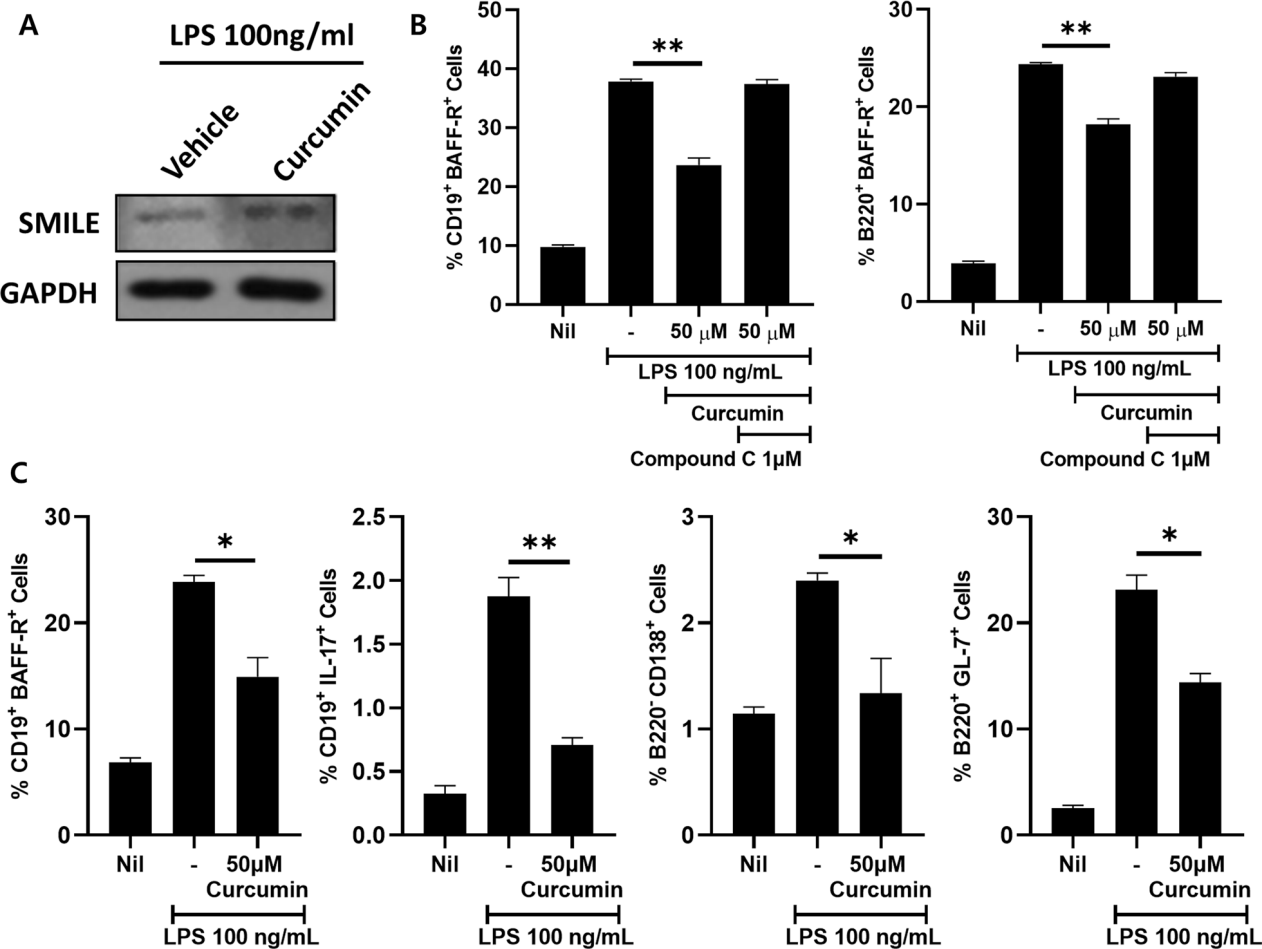
Effect of curcumin treatment on mice splenocytes in vitro. CIA mice splenocytes were stimulated with LPS in the presence of curcumin and/or compound C for 3 days and then analyzed by flow cytometry. **A** Western blots of SMILE expression in the splenocytes of curcumin (25 μM)-treated mice administered LPS (100 ng/mL) and untreated mice after 6 h. The SMILE expression was elevated in curcumin treated cells. **B** Expression of BAFF-R- and CD19-positive cells. Both cells were decreased by curcumin treatment, but curcumin had no effect in the group treated with the AMPK inhibitor compound C. **C** Proportion of BAFF-R^+^ B cells (CD19^+^ BAFF-R^+^), IL-17 secreting B cells (CD19^+^ IL-17^+^), plasma cells (B220^−^ CD138^+^), and GC B cells (B220^+^ GL-7^+^) after curcumin treatment. *P < 0.05 and **P < 0.01

As a previous study showed that curcumin increases the expression of SMILE, in this study curcumin was used as an inducer of SMILE [31–33]. The CIA mice splenocytes were cultured in the presence of 100 ng/mL of lipopolysaccharide (LPS) and treated with 50 μM curcumin. The results showed significant increases in the translation of SMILE (Fig. 6A). Curcumin treatment in the presence of 100 ng/mL of LPS reduced the population of CD19^+^ B cells and B220^+^ B cells expressing BAFF-R. However, in the group treated with the Compound C which is an AMPK inhibitor, the populations of CD19^+^ B cells and B220^+^ B cells expressing BAFF-R did not change. (Fig. 6B). BAFF-R-positive B (CD19^+^ BAFF-R^+^) cells, IL-17 secreting B (CD19^+^ IL-17^+^) cells, Plasma B (B220^−^ CD138^+^) cells and GC B (B220^+^ GL7^+^) cells proportion were decreased by curcumin treatment (Fig. 6C). This result showed that a curcumin-mediated increase in SMILE expression inhibits the development of B cells and suppresses the expression of BAFF-R in CD19^+^ B cells and CD220^+^ B cells through regulation of AMPK signaling pathway.

## Discussion

RA is a systemic autoimmune and inflammatory disease in which immune cells attack healthy cells and tissues [34]. Although RA does not affect survival, patients have painful swelling and joint destruction, which severely compromise their quality of life [35]. The risk factors for RA include age, sex, genetic factors, smoking, and obesity, but immune system dysfunction plays a major role [36–38]. Many RA studies have focused on T cells whereas relatively few similar studies have focused on B cells [39]. In a previous study, we showed that SMILE expression inhibits inflammatory bowel disease (IBD) in a mouse model of colitis via the regulation of Foxp3 expression [7]. In this study, we showed that autoimmune pathology in mouse models of RA (CIA and CAIA) was alleviated by the regulation of B cell activation through SMILE overexpression.

To investigate SMILE expression in RA patients, we searched public mRNA sequencing data and found that *CREBZF* expression was significantly reduced in our 10 RA patients compared to the levels in 10 healthy controls. This result suggested that the expression of SMILE, which is encoded by *CREBZF* [40], plays a critical role in RA development and immune cell changes.

We therefore investigated the therapeutic potential of SMILE in RA patients in an animal model by injecting CIA mice with a SMILE overexpression vector. In those mice, both the pathology score and the incidence of arthritis were reduced, consistent with the decrease in joint tissue damage. Immunohistochemistry in mouse joint tissues was then performed to identify the signaling pathways by which SMILE suppresses RA development. In a previous study we showed that SMILE expression leads to the upregulation of p-AMPK in mouse models of IBD and experimental autoimmune arthritis [7, 41]. SMILE overexpression was also shown to increase p-AMPK while decreasing mTOR and p-STAT3 expression. mTOR regulates the expression of genes that mediate inflammation, including NF-kB and STAT3 whereas AMPK is an upstream negative regulator of mTOR. BAFF-R levels were also elevated in SMILE-overexpressing mice. BAFF is secreted by macrophage or dendritic cells and plays a critical role in activating B cells by enhancing the levels of autoantibody-producing plasma cells and IL-6-producing effector B cells. BAFF also suppresses IL-10-secreting regulatory B cells. Taken together, this study demonstrated that SMILE expression has a regulatory effect on RA through the regulation of AMPK/mTOR signaling and BAFF expression.

The immunofluorescence images of mouse spleen tissues showed that the overexpression of SMILE notably decreased Th17 cells and increased Treg cells while reducing the number of plasma B cells. These data suggested SMILE-mediated changes in AMPK/mTOR signaling and BAFF expression leading to the regulation of immune cells in vivo. Although the change was not significant, SMILE overexpression decreased the levels of FOB cells and mature B cells and increased the levels of MZB cells, immature B cells, and regulatory B cells (CD5^+^ CD1D^high^ cells). These results demonstrate that the regulation of AMPK/mTOR and BAFF by SMILE modifies pro-inflammatory immune cells in vivo.

To verify the effect of SMILE, CAIA was induced in SMILE-overexpressing Tg mice. SMILE overexpression suppressed the tissue damage associated with CAIA and decreased collagen antibody production in tissues and blood. The immunofluorescence images indicated that the modifications in AMPK/mTOR and STAT3 induced by SMILE decreased the activation of B cells.

Curcumin, a member of the ginger family, is produced by plants of the *Curcuma longa* species [42, 43]. It is generally recognized as safe for its intended use by the U.S. Food and Drug Administration [44]. However, curcumin may also have therapeutic applications in numerous diseases [45]. Our previous studies showed an immune regulatory effect of curcumin in mouse models of autoimmune disease, although our focus was on T cell function [46, 47]. In other studies, curcumin has been shown to increase SMILE expression and regulate endoplasmic reticulum stress [32, 33]. Hence, in this study curcumin was used as a SMILE inducer. Curcumin was shown to elevate SMILE transcription by about fivefold, reduce B cell activation, and decrease the levels of plasma B cells, GC B cells, IL-17-secreting CD19^+^ B cells, and BAFF-R-expressing B cells in this data.

Despite the many studies of RA and its treatment, its pathogenesis has yet to be elucidated and there is no complete cure. In this study, we present the first evidence that SMILE can be used as a therapeutic agent for RA, based on its ability to inhibit B cell activation by regulating the AMPK/mTOR signaling pathway and BAFF expression.

## Data Availability

Requests to access the datasets should be directed to Mi-La Cho, iammila@catholic.ac.kr.
